# The Impact of Base Cell Size Setup on the Finite Difference Time Domain Computational Simulation of Human Cornea Exposed to Millimeter Wave Radiation at Frequencies above 30 GHz

**DOI:** 10.3390/s22155924

**Published:** 2022-08-08

**Authors:** Negin Foroughimehr, Zoltan Vilagosh, Ali Yavari, Andrew Wood

**Affiliations:** 1 School of Health Sciences, Swinburne University of Technology, Melbourne, VIC 3122, Australia; 2 Australian Centre for Electromagnetic Bioeffects Research, Swinburne University of Technology, Melbourne, VIC 3122, Australia; 3 School of Science, Computing and Engineering Technologies, Swinburne University of Technology, Melbourne, VIC 3122, Australia

**Keywords:** electromagnetic field, 5G telecommunication systems, cornea, Finite Difference Time Domain (FDTD) Method

## Abstract

Mobile communication has achieved enormous technology innovations over many generations of progression. New cellular technology, including 5G cellular systems, is being deployed and making use of higher frequencies, including the Millimetre Wave (MMW) range (30–300 GHz) of the electromagnetic spectrum. Numerical computational techniques such as the Finite Difference Time Domain (FDTD) method have been used extensively as an effective approach for assessing electromagnetic fields’ biological impacts. This study demonstrates the variation of the accuracy of the FDTD computational simulation system when different meshing sizes are used, by using the interaction of the critically sensitive human cornea with EM in the 30 to 100 GHz range. Different approaches of base cell size specifications were compared. The accuracy of the computation is determined by applying planar sensors showing the detail of electric field distribution as well as the absolute values of electric field collected by point sensors. It was found that manually defining the base cell sizes reduces the model size as well as the computation time. However, the accuracy of the computation decreases in an unpredictable way. The results indicated that using a cloud computing capacity plays a crucial role in minimizing the computation time.

## 1. Introduction

The Millimetre Wave (MMW) of Electromagnetic (EM) radiation is defined as radiation in the 30–300 GHz range. The high frequency and propagation characteristics make MMWs extremely attractive for 5G wireless communication systems and industrial and imaging applications. Emerging “5G and beyond” communication systems deliver data rates in the order of 100 Megabits to 20 Gigabits per second and lower latency for many simultaneous users. Given the increasing use of the MMW band in emerging telecommunication systems, it is important to determine the likelihood of adverse impacts on the human cornea. The diagnostic imaging applications of MMWs for in vivo assessment of corneal hydration have been also of great interest and is a subject of ongoing study [1,2].

The cornea consists of 78% of water. Water has a very high dielectric constant that is highly absorptive to high GHz and THz EM radiation. Hence, MMW and THz are sensitive to water content in the tissue. Moreover, the extreme homogeneity and lack of physiological variations of the cornea compared to other organs and tissue in the body allow high GHz and THz imaging to be a promising imaging modality in ophthalmology [2].

The dielectric properties of water in the 6–300 GHz range result in an absorption coefficient (at 20 ${}^{{}^{\circ}}$C) rising from 4 ${\mathrm{cm}}^{-1}$ at 6 GHz to 30 ${\mathrm{cm}}^{-1}$ at 30 GHz, 85 ${\mathrm{cm}}^{-1}$ at 100 GHz, and 150 ${\mathrm{cm}}^{-1}$ at 300 GHz [3,4]. The resultant penetration depth at 30 GHz is about 1 mm at 30 GHz down to about 0.3 mm at 100 GHz.

This feature results in the EM energy being mainly deposited superficially in high water content tissues [5]; thus, the resultant effects of the exposure on the outer layers of the skin and the eye become the most important factor in assessing biological effects. Exposure of the eyes results in the majority of the absorption of EM energy by surface layers of the cornea [6].

In recognition of the superficial nature of energy deposition, the International Commission on Non-Ionizing Radiation Protection (ICNIRP) and the Institute of Electrical and Electronics Engineers specifies exposures in this frequency band in terms of the absorbed power density ($S_{abs}$) and the incident power density, ($S_{inc}$) rather than Specific Absorption Rate (SAR) [7,8].

The Finite-Difference Time-Domain (FDTD) method has become a preferred method for conducting EM simulations for biological impacts from wireless devices [8]. FDTD offers no inherent limit to the size of a simulation; the practical limitations are the capacity of the computer system used to accommodate the number of voxel cells and the computing time. In effect, the capabilities of the computer system dictate the size of the model in terms of the number of voxel cells that can be studied in simulation. Models that can be studied on common desktop computers, assisted by cloud computing capacity, broaden access to dosimetry studies to less well-resourced groups. The question becomes: how much accuracy is gained in the study of biological systems exposure with a given decrease in the voxel cell size in the simulation? Is the accuracy at a voxel size of λ/15 sufficient, or is λ/60 or higher necessary for an accurate assessment of exposure? There is a trade-off; a smaller voxel cell size puts a limit on the size of the model, which means that only a smaller, less representative model can be accommodated in the computer’s random-access memory (RAM), since the model needs to be held in RAM for the simulation process.

A smaller model of the relevant tissue may reduce the accuracy of the modeling more than the change in the voxel cell size, since a representative region may not be able to be properly characterized. There is also the consideration of the timestep. A short duration timestep improves the accuracy of the simulation but increases simulation time.

The FDTD technique solves the differential time-domain Maxwell’s equation (Equations (1) and (2)), which means that the calculation of the EM field values progresses at discrete steps in time.

(1) $$\nabla \times \vec{E}=-\mu \frac{\partial \vec{H}}{\partial t}-\sigma_{M}\vec{H}$$

(2) $$\nabla \times \vec{H}=\varepsilon \frac{\partial \vec{E}}{\partial t}+\sigma \vec{E}$$

where ε and σ are the electric parameters and μ and $\sigma_{M}$ are the magnetic parameters of the material, respectively. In Yee’s scheme, the electric fields are cantered on the edges of the cell, while the magnetic fields are cantered on the faces, which is the basis of the FDTD method [9]. The timestep parameter is the time required for the field to travel from one cell to the next. The electric fields and then the magnetic fields are computed at each timestep in succession. The steps continue until the desired endpoint has been reached. The smaller the timestep, the more accurate the simulation. This factor is only relevant to the time required for the simulation, not the size of the model, since the capacity to undertake the simulation in terms of the total voxels cells is determined by the computer system RAM. The maximum timestep allowed in the simulation space is:

(3) $$\Delta t=\frac{1}{C}{\left(\frac{1}{\Delta x^{2}}+\frac{1}{\Delta y^{2}}+\frac{1}{\Delta z^{2}}\right)}^{-\frac{1}{2}}$$

where *c* is the speed of light, and Δx, Δy, and Δz are the lengths of the cell sides in meters.

Vilagosh et al. have examined absorption by the skin and ear canal using the same computational approach by implementing XFdtd numerical software [10,11]; hence, this study extends previous works in our group. The simulated voxel cell size, when expressed in terms of the wavelength, determines the accuracy of an FDTD simulation.

A high-resolution computational model of the human cornea has been developed in this study, which harvests large quantities of information and is capable of easy optimization. The model incorporates the features of a realistic cornea and surface hydration by including the tear film.

Since the cell sizes decrease in three dimensions, the computational demands on representational models increase as the cube of the frequency; a model at 100 GHz will have 37 times more voxel cells than one at 30 GHz. The question of how changing the voxel cell size (expressed in terms of the wavelength) influences accuracy was explored in a set of FDTD simulations. This paper outlines the detail of the elements of the Yee cell meshing and the dielectric properties of the ocular tissues.

## 2. Methodology

### 2.1. Configuration of Mathematical Model

XFdtd Bio-Pro (version 7.9.2., Remcom, State College, PA, USA) is a three-dimensional full-wave EM solver based on the FDTD method. The solver was used as the platform for the anatomical design and the computational implementation of the simulations.

The mathematical models are often limited by computational capability, necessitating oversimplified anatomy. The computational complexity and memory requirements of the FDTD algorithm solving Maxwell’s equations is discussed in [12].

In this study, XFdtd Bio-Pro was used utilizing a cloud computing environment (i.e., National eResearch Collaboration Tools and Resources platform, provided by Australian Research Data Commons. This allows the complexity and the variability of human ocular tissues to be better represented while decreasing the computation time. The current constructed model harvests large quantities of information and is capable of easy optimization and expansion.

The initial step for the FDTD approach is the discretization of space and time into the grid. Space is divided into cells, which are small compared to the wavelength. The simulation was undertaken using the radiation source at three frequencies (30, 60, and 100 GHz).

### 2.2. Anatomical Design

Given the nature of the problem, the direct study of human subjects is hazardous. Designing a mathematical model is a viable and attractive alternative to predicting the cornea’s temperature elevation due to MMW exposure.

The cornea is elliptical with non-identical vertical and horizontal diameters. The anterior horizontal diameter is 12 mm, and the anterior vertical diameter is 11 mm. If viewed from the posterior, the cornea appears circular, with horizontal and vertical diameters of 11.7 mm [13]. For the sake of simplicity, the cornea is assumed to lie above the anterior chamber in the form of a hemispherical shell with a constant diameter and thickness of 12 mm and 0.5 mm, respectively. The anterior surface of the cornea is covered by the tear film with a thickness of 10 μm. In the presented model, the anterior chamber is bounded anteriorly by the cornea and posteriorly by the iris. The anterior chamber is filled with aqueous humor that is assumed to be stagnant.

**Figure 1:**
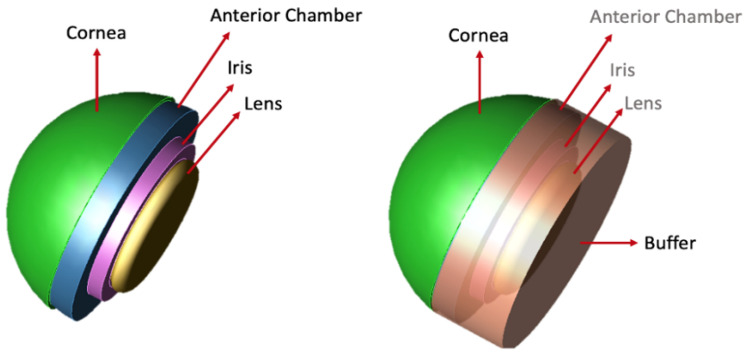
The constructed model of the anterior regions of the human. The model contains the tear film, cornea, anterior chamber (filled with aqueous humor), iris, and lens. The rear of the model contains a buffer cylinder to diffuse any reflected waves.

In the actual eye, the size of the pupil varies as a result of different lighting conditions. However, in this model, the pupil is assumed to have a constant diameter when the light intensity is low. The blood flow inside the choroid is one of the principal heat sources within the eye. Compared to the blood flow inside the choroid, the blood flow inside the iris and ciliary body are relatively small; thus, the thermal model presented in this study has ignored the presence of blood flow in this space. Finally, a buffer cylinder is placed at the model’s rear to diffuse reflected waves. For this purpose, the refractive index is defined to be the same as the back of the model, and the absorption coefficient is set to be higher to minimize internal reflection (shown in the last panel of Figure 1).

### 2.3. Dielectric Properties

In modeling the MMW interaction with the cornea, the dielectric and thermal properties of ocular tissues are presented in Table 1 and Table 2 respectively. The constructed model comprised six ocular tissues, with their dielectric properties directly gleaned from [14]. We have also considered the impact of temperature variation (from 20 ${}^{{}^{\circ}}$C to 35 ${}^{{}^{\circ}}$C). In this study, we used the dielectric properties of the ocular tissues at 35 ${}^{{}^{\circ}}$C. The conductivity (σ) of ocular tissues were calculated using Equation (4).

(4) $$\sigma =\varepsilon_{0}\varepsilon^{\prime \prime }2\pi f$$

where $\varepsilon_{0}$ is the permittivity of free space ($8.85\times 10^{-12}m^{-3}{\mathrm{kg}}^{-1}s^{4}A^{2}$), and *f* is the frequency of radiation.

The FDTD grid defines the E-field at the edges of cells. The Specific Absorption Rate (SAR) value is referenced to the center of the FDTD cell and is formed by summing the contributions of twelve E-fields on the edges of the FDTD cells.

SAR is the basic metric for specifying RF heating, which is the rate of energy transferred relative to the tissue mass in (W/kg) and is defined by Equation (5).

(5) $$SAR=\frac{\sigma {\left|E\right|}^{2}}{\rho }$$

where σ is the electrical conductivity (S/m), E is the maximum intensity of the E-field, and ρ is the material density, which is defined in ${\mathrm{kg}/m}^{3}$ in XFdtd. For temperature rise calculations in extended time, a modified form of bioheat equation (Equation (6)) is used [17].

(6) $$\Delta T_{t}=\frac{k_{t}\nabla^{2}T_{t}+\rho_{t}SAR-\rho_{b}c_{b}BP\left(T_{t}-T_{b}\right)}{\rho_{t}C_{t}}\Delta t$$

where $k_{t}$ is the thermal conductivity, $T_{t}$ is the tissue temperature, and $T_{b}$ is the blood temperature. $\rho_{t},C_{t}$, and BP is the blood tissue density, heat capacity of blood, and blood perfusion rate in the tissues, respectively. Since SAR and temperature rise are directly related [7], for thermal calculations involving short periods, it is sufficient to use the bioheat equation, where the contribution from blood flow, diffusion, and radiative loss are neglected (Equation (7)).

(7) $$\Delta T_{t}=\left(\frac{SAR}{C_{t}}\right)\Delta t$$

where $\Delta T_{t}$, $C_{t}$, and Δt are the temperature rise and heat capacity and the length of time, respectively. It is worth mentioning that this study forms one part of a large research project, and it particularly outlines the details of the EM solver and the meshing size setup in the model. The thermal properties of ocular tissues are currently being used for estimating the temperature rise in ocular tissues by solving the bioheat equation in our ongoing studies.

### 2.4. Exposure Scenario

The basis for the simulation is the construction of models that would represent the scenario where the anterior region of the eyeball is exposed to MMW at different frequencies. In XFdtd, an excitation source can be applied externally in the form of incident plane waves for scattering calculations or Gaussian beams for optical frequency calculations.

**Figure 2:**
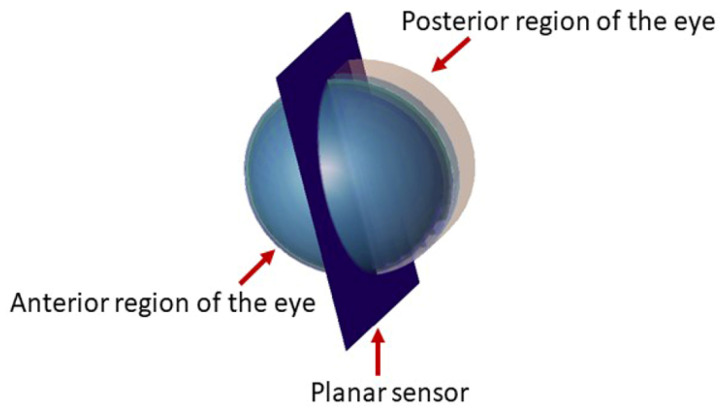
The placement of the planar sensor bisecting the model in the sagittal plane.

The radiation source is directed toward the anterior eye surface, perpendicular to the corneal eye cross-section. The excitation was set at 3.0 V/m in amplitude at 30, 60, and 100 GHz. A planar E-field sensor (which depicts the time variant absolute values of E-field at a given cutplane) was placed vertically, bisecting the model in the sagittal plane as illustrated in Figure 2. Planar sensors generated a series of false color images at different time intervals, illustrating the relative value of E field in dB. The sources of 60 GHz and 100 GHz were specified with the same computational setup to explore the relative value of the E-field after exposure to MMW.

### 2.5. Meshing Constraint

The simulation’s accuracy depends upon the ratio of the wavelength of excitation to the Yee cell (mesh size). Electric material properties are assigned to the appropriate cell edge during the meshing operation. For a homogeneous media, a rule of thumb value of λ/10 is commonly used for the maximum cell size [18]. In this study, we use finer resolutions, which provide better accuracy, but also require more computer resources.

**Figure 3:**
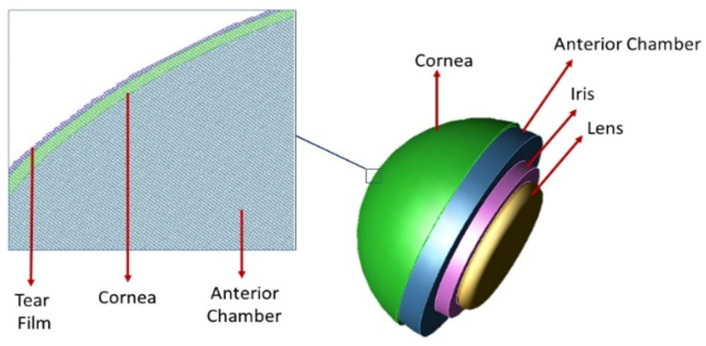
The model’s mesh cutplane illustrates the meshing priority; the tear film and the cornea have higher meshing priority than the aqueous humor in the anterior chamber.

XFdtd creates the mesh by examining where each piece of geometry in the simulation intersects the grid and applying the material assigned to that piece of geometry to the appropriate Yee cell edge and faces. Assigning meshing priority is a critical step in the meshing configuration. When tissues with different dielectric properties overlap, determining which dielectric properties should be applied to the mesh becomes difficult. To avoid sending a questionable mesh to the calculation engine for simulation, the meshing priority was given to the tear film and the cornea as shown in Figure 3. In addition, the specification of absorbing boundary plays a crucial role in the accuracy of the FDTD method [19]. The absorption type was set as a Perfectly Matched Layer (PML) for all the simulations.

#### 2.5.1. Specifying Cell Sizes Manually

The cell sizes were manually specified in the Grid editor. In XFdtd, PrOGrid Project Optimized Gridding is a set of Gridding tools that simplify the process of finding the optimal trade-off between runtime cost and accuracy. When the PrOGrid is unchecked, the base cell sizes can be entered manually for each Cartesian direction. This was done to compare the variation in cell sizes and their impact on the duration and the accuracy of the simulations. As noted previously, when defining the cell size for a Grid region, it is essential to specify the cell size much less than the smallest wavelength. Various target cell sizes in the range of 18 to 60 cells per wavelength are manually specified to resolve small geometric features.

#### 2.5.2. Specifying Cell Sizes in Adaptive (PrOGrid) Mode

For adjustments of the mesh properties for individual ocular tissues, the automatic cell size mode was used (i.e., PrOGrid Project Optimized Gridding). This option enables parts with electric and magnetic materials, other than free space, to be included in the mesh. The PrOGrid option increases grid resolution in high permittivity dielectrics. When this option is used, the base cell sizes are determined from the project’s frequency range of interest and the minimum cells per wavelength setting. The values of 18, 25, 30, and 60 were chosen for the minimum cells per wavelength.

## 3. Results

### Choosing an Appropriate Grid Size Constraint

To ensure that accurate results are obtained, the defined target cell size for a grid region needs to be significantly smaller than the smallest wavelength.

A commonly applied constraint is ten cells per wavelength, meaning that the length of any grid edge should be less than one-tenth of the wavelength at the highest frequency (i.e., shortest wavelength) of interest. It is necessary to consider a smaller FDTD grid cell size to provide adequate geometry resolution [20].

As noted earlier, the mesh density (grid cell size) has a major impact on the accuracy of the results. There is an exponential growth of model size and computing time with decreasing mesh cell size. The changes associated with variations in grid cell size in the constructed human eye model are illustrated in Table 3 and Table 4 for the manual and PrOGrid cell size setup, respectively. The model’s size represents the estimated total system RAM requirement for CPU-based simulations.

**Figure 4:**
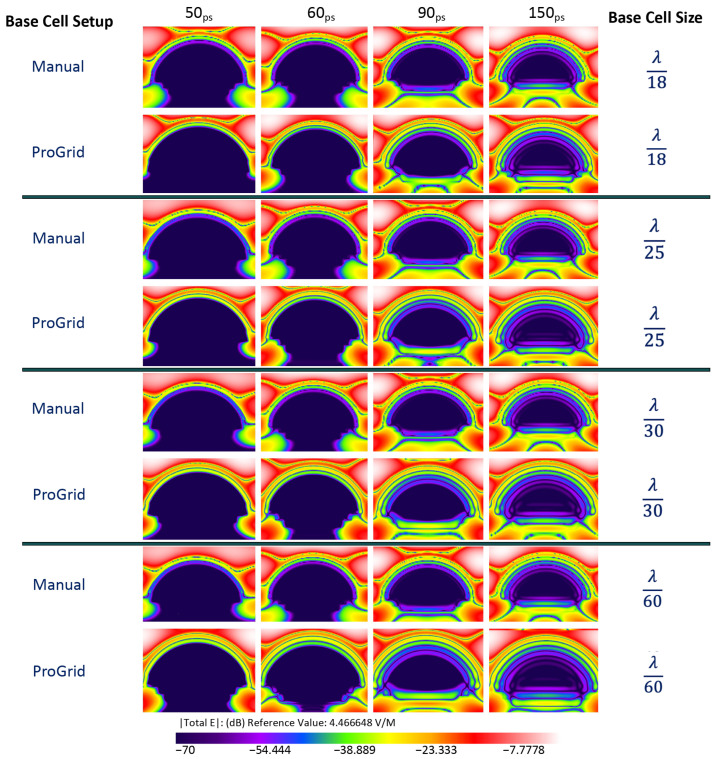
The output of planar E-field sensors for an EM source of 30GHz; the location of a planar E-field sensor placed vertically within the cornea. The simulation was carried out for λ/18 and λ/25, λ/30, and λ/60 using manual and PrOGrid setup.

**Figure 5:**
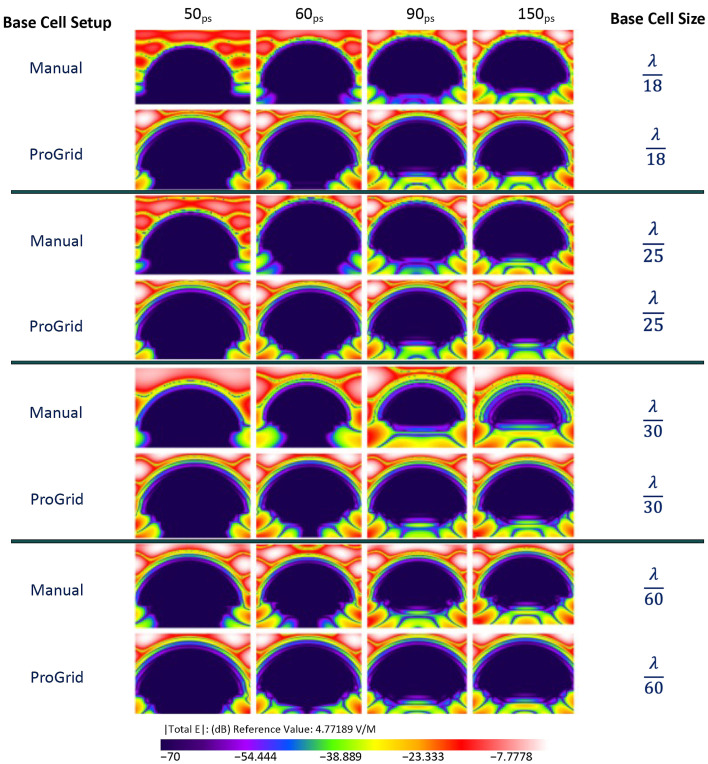
The output of planar E-field sensors for an EM source of 60GHz; the location of a planar E-field sensor placed vertically within the cornea. The simulation was carried out for λ/18 and λ/25, λ/30, and λ/60 using manual and PrOGrid setup.

**Figure 6:**
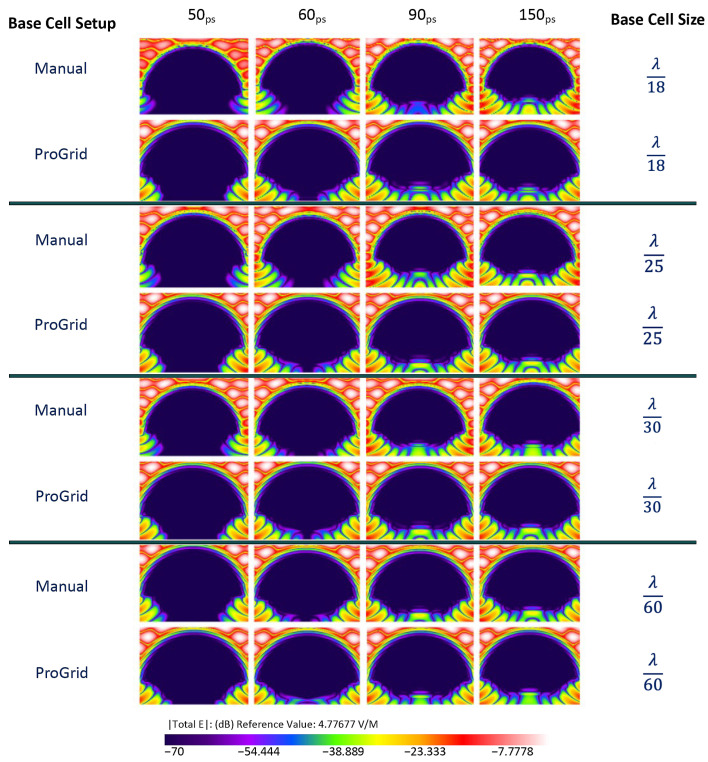
The output of planar E-field sensors for an EM source of 100GHz; the location of a planar E-field sensor placed vertically within the cornea. The simulation was carried out for λ/18 and λ/25, λ/30, and λ/60 using manual and PrOGrid setup.

**Figure 7:**
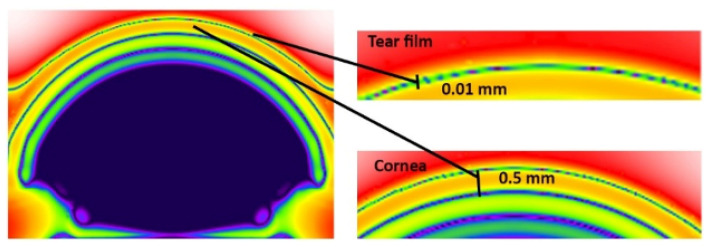
The detail of distribution of the E-field (in dB) in the anterior part of the eye model.

Each simulation was run for 10,000 timesteps. The planar sensor begins to gather data approximately at 7 ps into the simulation. Numerical results are shown in Figure 4, Figure 5 and Figure 6 presents the E-field distribution for 30 GHz, 60 GHz, and 100 GHz radiation sources using various voxel sizes. The output of the planar sensor at 50, 60, 90, and 150 ps is depicted as a 10 discrete color, −70 to 0 dB display. The detail of distribution of E-field in dB in the anterior region of the eye is depicted in Figure 7.

**Figure 8:**
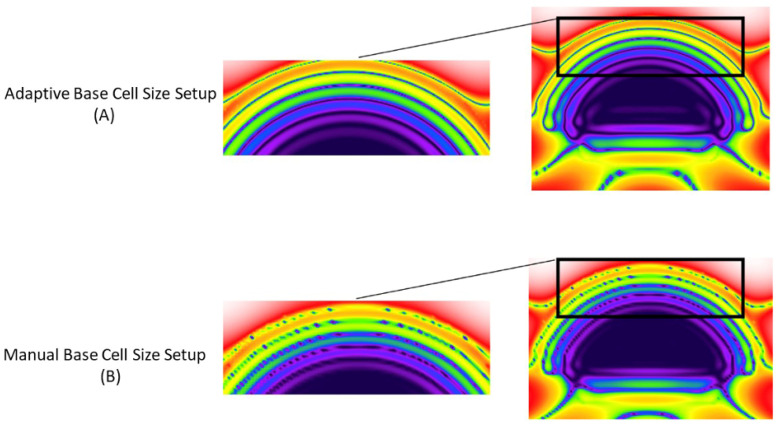
The output of planar sensor at 150 ps, using a 30 GHz radiation source. The sensor depicts the detail of distribution of the E-field in dB in the anterior region of the eye model. The paired images show the same voxel size (λ/18 and λ/25) using two different techniques of the base cell size specification: adaptive and manual cell size setup on the top (**A**) and bottom (**B**), respectively.

**Figure 9:**
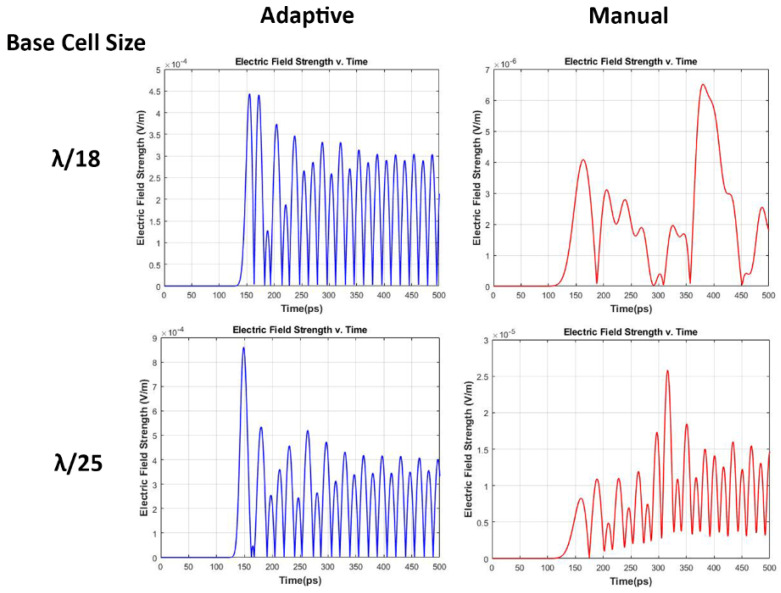
The output from the point sensor at the surface of the cornea. By using adaptive cell size specification, the initial peak of the E-field strength is within the same range using different cell sizes (λ/18 top and λ/25 bottom).

The XFdtd enables precisely determining the E-field distribution within biological tissues. In this study, the computational accuracy is assessed by comparing the E-field distribution in the eye model, which is shown in Figure 8. The field distribution depends on the intensity of the radiation source as well as the characteristic of each tissue. As illustrated in Figure 8A, the E-field is unequally distributed within the eye following an excitation of 30 GHz. The unequal E-field distributions are due to different characteristics of various ocular tissues. Moreover, we measured the change over time of the absolute values of the E-field using a point sensor. The output from a point E-field sensor in Vm${}^{-1}$ is displayed graphically in Figure 9 as a set of time versus field strength data using different cell size specification techniques. The point sensor output of manual cell size setup reveals inconsistent E-field strength behavior at the early stages of the movements of the wave-front.

## 4. Discussion

**Figure 10:**
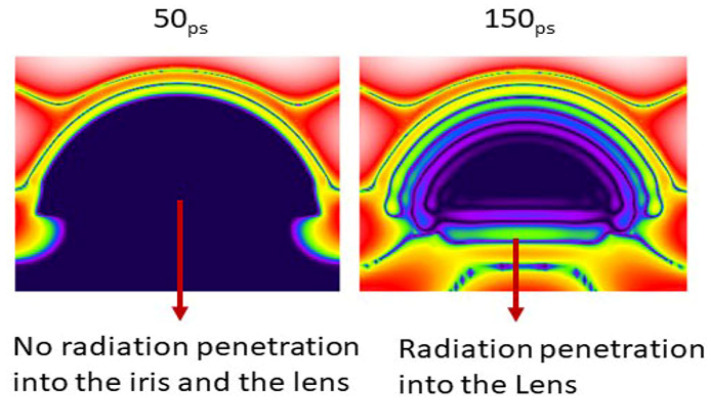
Radiation penetration and exposure pattern at the internal eyeball structures as time progresses; an excitation source of 30 GHz is applied.

Analysis of the computational results have shown that EM radiation is highly absorbed by anterior eye tissues (e.g., cornea and the tear film). The possibility of substantial penetration of MMW radiation of 30–100 GHz radiation into the ocular tissues cannot be ignored (Figure 10). Computational simulations are useful as an initial investigation; however, they require being accurate in terms of the computational setup, specifically when calculating the temperate elevations within ocular tissues.

When the PrOGrid (adaptive mode) is on, the minimum cells per wavelength, as well as the wavelength of the project’s frequency of interest, set the base cell size of the grid. Increasing the value of minimum cell per wavelength reduces cell edge length, thus increasing accuracy and computation time. It is worth mentioning that the size of the smallest geometric feature in the project is crucial to resolve, which is known as Minimum Feature Size in the XFdtd PrOGrid option. This is automatically defined when the PrOGrid is checked, which significantly enhances the accuracy of the result. Using the automatic base cell size setting provides reasonable grid cell sizes for the range of frequencies being simulated. It also increases grid resolution in high permittivity dielectrics and captures small geometric features with multiple grid cells. However, this option consumes approximately 10 times CPU time and 40 times RAM when defining smaller cell sizes. For instance, halving the cell size for a radiation source of 30 GHz increases memory requirements 4.9-fold, resulting in a 3.5-fold increase in the computation time.

The manual cell size setup, on the other hand, shows a considerable decline in the computation time as well as the required computer resources. The detailed distribution of the E-field in the anterior region of the eyeball (Figure 8) indicates that manually setting up the base cell sizes results in artifact formation of reflections and hotspots. The smooth E-field distribution was observed by a planar sensor using an adaptive grid specification. In line with the planar sensor data, the point sensor within the cornea using a manual cell size setup showed an unrealistic behavior of E-field strength vs. time compared with the adaptive cell size specification (Figure 9). This represented a substantially lower value of E-field strength when defining manual grid cell sizes.

When using an adaptive PrOGrid option, small geometric features are captured with multiple grid cells. It seems that manually setting up the cell sizes results in unwanted radiation artifacts, making the simulations inaccurate (Figure 8B). This could be due to coarse cell sizes, which fail to resolve critical geometric features within the eye model.

Choosing a cell size in the range of twenty cells per wavelength is a good starting point for the current model where small cells are required to resolve small geometric features (i.e., the cornea). Reducing the cell sizes are always at the expense of additional runtime and simulation memory. A smaller cell size such as λ/60 will require more computer resources, drastically increasing the computational time (e.g., approximately seven-fold in the computation time compared to λ/18 for a radiation source of 30 GHz). Thus, it may not be worth specifying very small cell sizes in the range of sixty cells per wavelength, specifically when using a desktop computer since for the computational simulation to be practical in an ordinary research setting, the size of the model needs to be accommodated on available computer resources.

Since the FDTD method calculates the E-field and H-field at every cell in the whole computational problem space at every timestep, the current constructed model has the capacity to have multiple sensors (e.g., planar and point sensors) inside or outside the model itself. The only limitation of this method is the data storage capacity and the computational time. The computational resource estimations underline the fact that this model with multiple ocular layers is not potentially suitable for desktop computer users.

As mentioned earlier, defining accurate base cell sizes for reasonable computations using the FDTD technique requires a high-end computer with several hundred megabytes of computer memory. In this study, the largest simulation with a multi-layer eye model took approximately 153 min using 32 processing cores and 128 GB of memory in a cloud computing environment.

The E-field distribution calculation required much higher computation time (i.e.,10-fold) using a desktop computer with six processing cores and 48 GB of memory, as compared to a cloud computing environment with 32 processing cores and 128 GB of memory (Table 5).

According to our numerical simulation, using a cloud computing environment with more processing cores and memory can be helpful in reducing the computation time for studying biological effects. Additionally, using an adaptive cell size setup is an effective approach for obtaining accurate computational results.

## 5. Conclusions

In this study, the human cornea with realistic anatomical layering was simulated using an FDTD solver (XFdtd) to assess the MMW absorption in the anterior region of the eyeball. This study focused on assessing the impact of grid (cell) size on the E-field distribution and E-field strength vs. time. Defining smaller grid sizes leads to more accurate results. However, the computational efficiency is substantially impacted by the need to utilize the timestep corresponding to smaller grid sizes throughout the simulation space. The results of presented simulations suggest that using an adaptive cell size specification (i.e., PrOGrid Project Optimized Gridding) provides fewer radiation artifacts, resulting in more accurate computational simulations.

Furthermore, we found that adaptive cell size setup radically increased the required computation time compared to manually specifying the cell sizes. We have utilized a cloud computing environment to process the simulations in an appropriate amount of time.

To sum up, using an adaptive cell size specification proved to be not only useful at lower frequencies, but also practical for biological studies at higher frequencies.

A future research direction for this paper will be an in vivo experimental evaluation of the result obtained from the computational model discussed in this paper.

## Figures and Tables

**Table 1:** The values used for the real ($\varepsilon^{\prime }$), imaginary ($\varepsilon^{\prime \prime }$) parts of complex permittivity, and conductivity (σ) at 35 ${}^{{}^{\circ}}$C for the ocular tissues used in the simulation [14].

Frequency	Tissue	$\varepsilon^{\prime }$	$\varepsilon^{\prime \prime }$	$\sigma \frac{S}{m}$	Refractive Index	Absorption Coefficient (cm${}^{-1}$)
30 GHz	Cornea	19.5	20.3	33.86	4.88	26
	Aqueous humor	32.1	33.8	56.38	6.27	34
	Iris	20.4	25.7	42.87	5.16	31
	Lens	16.7	20.20	33.70	4.63	27
60 GHz	Cornea	10.5	12.8	42.71	3.68	44
	Aqueous humor	15.3	24.4	81.41	4.70	65
	Iris	6.02	18.1	60.39	3.54	64
	Lens	6.77	13.6	45.37	3.31	52
100 GHz	Cornea	8.26	7.54	41.93	3.12	51
	Aqueous humor	9.74	16.6	92.31	3.81	91
	Iris	3.16	8.98	49.93	2.52	75
	Lens	3.98	7.78	43.26	2.52	65

**Table 2:** The thermal properties of ocular tissues used in the FDTD simulations [15,16].

Ocular Tissue	Thermal Conductivity K W/(m·K)	Specific Heat Capacity C J/(kg·K)	Density ρ (kg/m${}^{3}$)	Blood Perfusion W/(m${}^{3}$·K)
Cornea	0.58	4178	1050	-
Aqueous humor	0.58	3997	996	-
Iris	0.52	3600	1050	3500
Lens	0.4	1000	3000	-
Water	0.57	4180	1000	-

**Table 3:** The changes associated with variations in cell size and the computation time using manual cell size setup. The lower frequencies have a larger wavelength and for a given wavelength, the minimum cell per wavelength is larger in absolute terms.

Frequency	Min Cell per Wavelength	Base Cell Size (mm)	Model Size	Timestep Duration	Total Computation Time (Seconds)
30 GHz	18	0.555171	92.1 MB	7.41444 × 10${}^{-13}$	51
	25	0.399723	105 MB	5.37175 × 10${}^{-13}$	57
	30	0.333103	116.1 MB	4.48187 × 10${}^{-13}$	547
	60 t	0.166551	222.5 MB	2.23959 × 10${}^{-13}$	550
60 GHz	18	0.277568	132.2 MB	3.71732 × 10${}^{-13}$	29
	25	0.199862	179 MB	2.68301 × 10${}^{-13}$	38
	30	0.166551	221.1 MB	2.23959 × 10${}^{-13}$	59
	60	0.083275	678.7 MB	1.11927 × 10${}^{-13}$	148
100 GHz	18	0.166551	222.1 MB	2.23959 × 10${}^{-13}$	47
	25	0.119917	356.3 MB	1.61279 × 10${}^{-13}$	109
	30	0.099930	484.4 MB	1.34474 × 10${}^{-13}$	150
	60	0.049965	2 GB	6.72217 × 10${}^{-14}$	388

**Table 4:** The exponential growth of model size and computation time with reducing mesh cell size (cell sizes were specified in PrOGrid mode).

	30 GHz		60 GHz		100 GHz	
Min Cells per Wavelength	Model Size	Total Simulation Time (s)	Model Size	Total Simulation Time (s)	Model Size	Total Simulation Time (s)
18	616.5 MB	909	849.2 MB	876	1.5 GB	1471
25	1.2 GB	1476	1.6 GB	1007	3.1 GB	1503
30	1.8 GB	1768	2.5 GB	1171	4.8 GB	1546
60	8.9 GB	6301	13.1 GB	4395	27.5 GB	9179

**Table 5:** The comparison of computation time using an ordinary desktop computer and a cloud environment.

Frequency (GHz)	Min Cells per Wavelength	Total Simulation Time (s) (Desktop)	Total Simulation Time (s) (Cloud Environment)
30	25	10,097	1476
60	25	10,616	1007
100	25	16,069	1503
